# The Epidemiology of Rift Valley Fever in Mayotte: Insights and Perspectives from 11 Years of Data

**DOI:** 10.1371/journal.pntd.0004783

**Published:** 2016-06-22

**Authors:** Raphaëlle Métras, Lisa Cavalerie, Laure Dommergues, Philippe Mérot, W. John Edmunds, Matt J. Keeling, Catherine Cêtre-Sossah, Eric Cardinale

**Affiliations:** 1 Centre for the Mathematical Modelling of Infectious Diseases, Department of Infectious Disease Epidemiology, London School of Hygiene & Tropical Medicine, London, United Kingdom; 2 UMR CMAEE, CIRAD, Sainte-Clotilde, La Réunion, France; 3 UMR1309 CMAEE, INRA, Montpellier, France; 4 Bureau de la Santé Animale, Direction Générale de l’Alimentation, Paris, France; 5 Université de La Réunion, St Denis, France; 6 GDS Mayotte-Coopérative Agricole des Eleveurs Mahorais, Coconi, Mayotte, France; 7 Direction de l’Alimentation, de l’Agriculture et de la Forêt de Mayotte, Mamoudzou, France; 8 WIDER, Warwick University, Coventry, United Kingdom; 9 Life Sciences, Warwick University, Coventry, United Kingdom; 10 Mathematics Institute, Warwick University, Coventry, United Kingdom; School of Veterinary Medicine University of California Davis, UNITED STATES

## Abstract

Rift Valley fever (RVF) is a zoonotic arboviral disease that is a threat to human health, animal health and production, mainly in Sub-Saharan Africa. RVF virus dynamics have been poorly studied due to data scarcity. On the island of Mayotte in the Indian Ocean, off the Southeastern African coast, RVF has been present since at least 2004. Several retrospective and prospective serological surveys in livestock have been conducted over eleven years (2004–15). These data are collated and presented here. Temporal patterns of seroprevalence were plotted against time, as well as age-stratified seroprevalence. Results suggest that RVF was already present in 2004–07. An epidemic occurred between 2008 and 2010, with IgG and IgM peak annual prevalences of 36% in 2008–09 (N = 142, n = 51, 95% CI [17–55]) and 41% (N = 96, n = 39, 95% CI [25–56]), respectively. The virus seems to be circulating at a low level since 2011, causing few new infections. In 2015, about 95% of the livestock population was susceptible (IgG annual prevalence was 6% (N = 584, n = 29, 95% CI [3–10])). Monthly rainfall varied a lot (2–540mm), whilst average temperature remained high with little variation (about 25–30°C). This large dataset collected on an insular territory for more than 10 years, suggesting a past epidemic and a current inter-epidemic period, represents a unique opportunity to study RVF dynamics. Further data collection and modelling work may be used to test different scenarios of animal imports and rainfall pattern that could explain the observed epidemiological pattern and estimate the likelihood of a potential re-emergence.

## Author Summary

Rift Valley fever (RVF) is a viral zoonotic disease mainly present in Sub-Saharan Africa, transmitted by mosquitoes and primarily affecting livestock (cattle, sheep and goats). The epidemiology of the disease is not fully known, mainly because of data scarcity. In Mayotte, an island close to Madagascar, RVF has been present since at least 2004, and retrospective and prospective surveys have been conducted to collect data from livestock over 11 years. Our work uses this 2004–15 dataset to describe the past and current epidemiology of RVF on the island. Results suggest that the disease was endemic between 2004–07. An epidemic occurred between 2008 and 2010. Since 2011, the virus appears to be circulating at a low-level causing few new infections. Modelling work may be used to explain the observed epidemiological pattern, and estimate the likelihood of a potential re-emergence.

## Introduction

**Fig 1:**
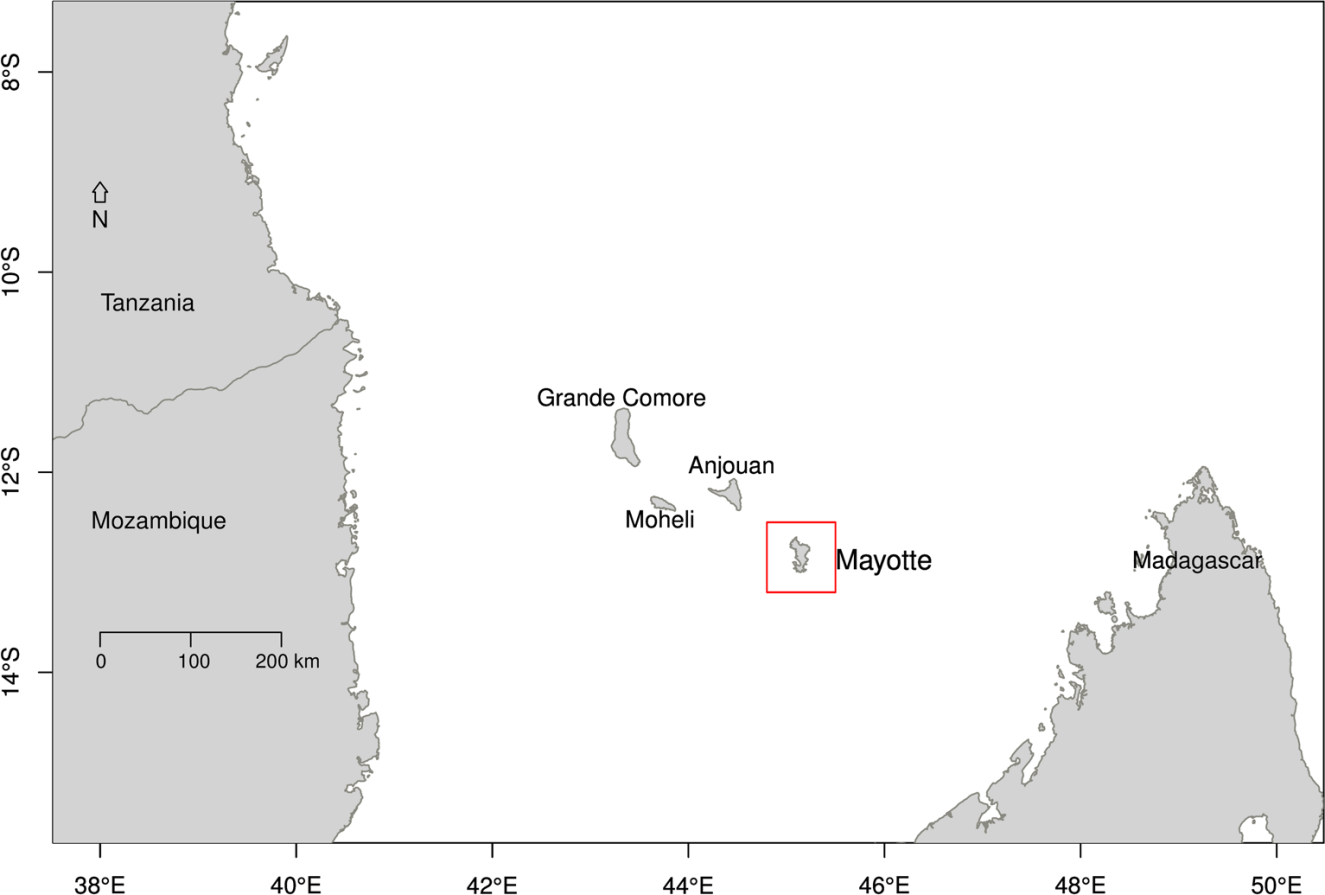
Location of the island of Mayotte in the Comoros archipelago in the Mozambique Channel (between Mozambique and Madagascar), off the Southeastern African coast. Mayotte is a French department, whilst Grande Comore, Moheli, and Anjouan, belong to the Union of the Comoros.

Rift Valley fever (RVF) is a zoonotic arboviral disease (*Phlebovirus*, Family *Bunyaviridae*) primarily affecting domestic livestock (cattle, sheep and goats). Epidemics of RVF in livestock mainly cause abortions and neonatal deaths. In humans, symptoms are usually non-specific causing an influenza-like syndrome, but sometimes infection can also result in meningo-encephalitis, haemorrhagic fever and death [1]. Since its first description in Kenya in 1931 [2], RVF has been reported in Sub-Saharan Africa, as well as in Egypt (1977), in Madagascar (1979), in the Arabian Peninsula (2000), and in the islands of the Comoros archipelago (2007) [3]; the latter being located in the North of the Mozambique Channel, between Mozambique and Madagascar (Fig 1).

RVF is transmitted to mammals mainly by mosquito bites (main vectors belong to the genera *Aedes* and *Culex*). The hypotheses underlying RVF virus emergence is the concomitance of *(i)* the presence of susceptible livestock, *(ii)* an increase in vector abundance (e.g. due to heavy rainfall) thereby facilitating virus amplification and transmission, and *(iii)* the presence of the virus itself. The virus may be imported through the movements of infectious animals or arthropod vectors, or be present locally. RVF virus may persist between epidemics at a low level in livestock causing sporadic cases, or be maintained in potential local reservoirs. The latter includes vertical transmission in mosquito eggs and a large range of wild mammalian hosts, such as buffaloes, rodents and bats, although no good evidence exists on the latter hypothesis [4–6]. Humans may acquire the virus mainly by contact with infectious animal tissues, and also possibly via mosquito bites [1].

In Mayotte, RVF virus was detected for the first time in 2007 in humans [7,8]. Sequencing related this virus to the 2006–2007 eastern African Kenya-1 lineage [9], suggesting a recent import onto the island from the African mainland. Retrospective serological analyses conducted on livestock sera (collected between 2004 and 2008) showed that RVF virus had been on the island at least since 2004, so before the 2007 introduction, but no virus sequencing had been done [10]. Since 2009, an on-going disease surveillance system (SESAM, Système d'Epidémiosurveillance Animale à Mayotte) has been monitoring livestock health status, and collecting livestock sera.

This paper aims at presenting eleven years (2004–15) of RVF serological data, merging the 2004–08 dataset presented in Cetre-Sossah et al. [10], the 2012–13 dataset presented in Cavalerie et al. [11], with all other serological analyses conducted between 2008 and 2015. This large dataset collected on an insular territory represents a unique opportunity to study RVF epidemiology. It will be used to describe the past and current RVF status in Mayotte and propose relevant further data collection and mathematical modelling work to study RVF virus dynamics.

## Methods

### Study site

The island of Mayotte is a French overseas department that belongs to the Comoros archipelago (Fig 1). It is a small island of 374km^2^ (about 35km North-South and 10km East-West), with a high population density (212,600 inhabitants in 2012 [12]). The estimated livestock population is approximately 20,000 cattle and 13,000 small ruminants (sheep and goats). Average herd size is rather small (5 animals); with animals mainly kept outdoors year round and raised for family consumption or cultural ceremonies [13,14].

### Livestock data

Livestock (cattle and small ruminants) serological data were collated from different sources. The 2004–08 data were retrospective serological surveys [10]; whilst prospective data collections were conducted in 2008–15. The different surveys collated and the serological testing data are presented in Table 1, and detailed hereinafter.

**Table 1:** Rift Valley fever serological surveys conducted in cattle and small ruminants in Mayotte in 2004–15, number of sera tested and ELISA tests performed.

Survey	Description	Date	Species	No. sera tested	ELISA tests	Reference
1	Retrospective cross-sectional	2004	Cattle	130	IgG	[10]
2	Retrospective cross-sectional	2005	Cattle	130	IgG	[10]
3	Retrospective cross-sectional	2006	Cattle	126	IgG	[10]
4	Retrospective cross-sectional	2007	Cattle	129	IgG	[10]
5	Retrospective cross-sectional	May 2007—Apr 2008	Cattle	289	IgG	[10]
6a	Retrospective cross-sectional	Nov 2007—Mar 2008	Illegally imported goats	29	IgG	[10]
			Illegally imported goats	5	IgM	[10]
6b	Retrospective cross-sectional	Mar 2008	Cattle living close to illegally imported goats	79	IgG	[10]
			Cattle	12	IgM	[10]
6c	Retrospective cross-sectional	Mar 2008	Cattle	78	IgG	Data presented here
			Cattle	16	IgM	Data presented here
7a	Longitudinal 1	Jun 2008	Cattle and goats	273	IgG	[10]
7b	Longitudinal 2	Sept 2008	Cattle and goats	76	IgG	[10]
7c	Longitudinal 3	Feb 2009	Cattle and goats	79	IgG	[10]
8	Repeated cross-sectional	May 2009 –Jun 2015:		4565 (2550 animals)	IgG	
	(SESAM)			1497 (1204 animals)	IgM	
		1. May 2009 –April 2013	Cattle and goats	2410 (1297 animals)	IgG	[11]
				336 (244 animals)	IgM	Data presented here
		2. May 2013- Jun 2015	Cattle and goats	2155 (1653 animals)	IgG	Data presented here
				1161 (979 animals)	IgM	Data presented here

#### Serological testing

All sera were tested for IgG (ID Screen RVF Competition ELISA (IDVet, Grabels, France)) or IgM (ID Screen RVF IgM Capture ELISA (IDVet, Grabels, France)) antibodies by ELISA. For RVF, IgG antibodies are detectable in the blood of infected animals approximately 10 days post-infection (PI), and are believed to remain detectable for several years PI. IgM are detected earlier (approximately 5 days PI), but remain detectable for only approximately 3 months PI [1]. Therefore, the presence of IgG indicates animals ever infected, whilst IgM indicates those recently infected.

#### Retrospective cross-sectional serological surveys 2004–08

These surveys (surveys 1–6, Table 1) were carried out in 2008, when RVF virus was isolated in humans. They aimed to determine whether RVF was also or had previously been present in livestock, tracing back as far as possible. Little detail is available on how animals were selected in all those surveys; therefore we considered these as convenience sampled. Surveys 1–6 are ordered in Table 1 based on the date of animal sampling. The specific order in which these surveys were conducted is however know, presented in *Cetre-Sossah et al*. *2012* [10] and reminded below, together with the maximum information available.

The first retrospective cross-sectional surveys were surveys 6a-c conducted in March 2008 (Table 1). These included 29 illegally imported goats (survey 6a), 79 native cattle from the island living close to these goats (survey 6b), and additional 78 cattle sampled from other areas of the island (survey 6c). Whilst surveys 6a and 6b were targeted surveys in specific areas exposed to receiving illegally imported animals, survey 6c tested animals from different areas of the island. All sera were tested for IgG, and a subsample (26 sera chosen randomly) was also tested for IgM, to look for recent infections.

The second retrospective survey (survey 5) aimed at further exploring the geographical extent of past RVF infections, and analysed sera collected between June 2007 and May 2008, from herds sampled across the island. Finally, surveys 1–4 explored whether RVF had been present in Mayotte before the introduction of the 2006–2007 eastern African Kenya-1 lineage. These tested sera were randomly selected among sera collected for brucellosis prophylaxis (between 2004 and 2007), which were stored at the Veterinary Services offices. For surveys 1–5, sera were tested for IgG only.

#### Prospective serological surveys 2008–15

The first longitudinal study was conducted in small ruminants between June 2008 and February 2009. A total of 272 animals were sampled on the first occasion in June 2008, from 13 herds. These herds were selected across the island. Three months later, in September 2008, 76 animals were resampled, drawn only from herds negative in June 2008; and 79 animals from these herds were sampled again in February 2009 (surveys 7a, 7b and 7c in Table 1) [10].

An animal disease surveillance system was created in 2009 (SESAM, survey 8 in Table 1), whose target population was the livestock of Mayotte, and the source population (or sampling frame) was the official farm registry of the Chambre de l’Agriculture, de la Pêche et de l’Aquaculture de Mayotte (CAPAM). SESAM has been sampling livestock across the island selected from that list, based on farmers’ accessibility, agreement to be visited, and availability on the day of visit. Farms have been visited once or several times, with no strict regular temporal pattern due to resources and logistical reasons. Animals present on the farm on the day of the visit were sampled for RVF (and other diseases). Most importantly, animals were sampled regardless of their RVF status at the previous visit. Therefore, the design of data collection resembles a repeated cross-sectional design. Between May 2009 and June 2015, a total of 4565 blood samples from 2550 animals (75% cattle and 25% small ruminants, and about 70% females and 30% males) and 194 herds have been tested for RVF antibodies. The mean herd size was 9 (median = 7, interquartile range IQR = [4–12]). From these 4565 blood samples, all were tested for IgG only, and 32.8% (n = 1497) were tested for both IgG and IgM. IgM testing was randomly done on a subsample of sera when funding was available. IgM testing was independent of the IgG serology results.

#### Ethics statement

The studies were implemented with the approval of the Direction of Agriculture, Food and Forestry (DAAF) of Mayotte. Before 2015, animal sampling in this study was not subject to the approval of ethics committee nor to specific national of international regulations at the time of sample collection. Consent for blood sampling on a herd was obtained from its owner verbally after information in French (official language) or Shimaore (local language) was given. The animals were bled without suffering. No endangered or protected species were involved in the survey. For the 2015 data, all procedures on living animals were approved by the London School of Hygiene Animal Welfare and Ethical Review Board.

### Climate data

Rainfall and temperature are known to drive vector abundance and competence. The climate in Mayotte is marine tropical, with little temperature variation (average year round 24–34°C), but important rainfall (1500mm on average per year). Two main seasons are observed: hot rainy (December to March), and dry cool (June to September) [15]. In order to compare serological results to rainfall and temperature, data from 2004 to 2015 were sourced [16]. Monthly total rainfall and average temperature values were plotted against time, together with their average values by calendar month.

### Data processing and descriptive statistics

The total dataset included the results of 5720 samples from 3529 animals (from 448 herds, average herd size = 7.4, median = 5, IQR = [2–10]), sampled from and collected over 11 years (October 2004 to June 2015). All 5720 blood samples were tested for IgG, and 26.5% (n = 1513) were tested for both IgG and IgM. The dataset used the data from surveys 1 to 5, 6c, 7a and 8 (Table 1), defined as cross-sectional or repeated cross-sectional studies. Surveys 7b and 7c (the follow-up of the longitudinal study, totalling 155 sera) were not included as they clearly indicated measures of disease incidence; neither survey 6a nor 6b, as they were both targeted surveys. Due to the small size of Mayotte, estimates were produced for the whole island, as a single spatial epidemiological unit.

#### Temporal pattern of RVF seroprevalence

To look for the potential variation of RVF seroprevalence over the 2004–15 period, monthly and annual IgG and IgM prevalence estimates were plotted against time. For both IgG and IgM, monthly and annual prevalence were estimated as the number of RVF positive animals per month (or year) divided by the number of animals tested in that month (or year), accounting for clustering using the one-stage cluster sampling method varbin, *from* the aod R package [17]. When an animal was sampled more than once per year, it was counted once in the annual estimate, being classified as positive if at least one test was positive, and negative otherwise. Annual seroprevalence estimates were obtained by aggregating data from July of year *y* to June of year *y+1*. This temporal aggregation (named hereinafter “epidemiological year”) allowed estimating annual prevalences by capturing each rainy season as a whole, as opposed to aggregating by calendar year.

#### Age-stratified IgG prevalence

The livestock national identification database implemented in 2008 [14] provided the dates of birth for approximately 53% (n = 1346 animals) of the sampled animals from survey 8. To get insight on RVF dynamics through time, age-stratified IgG prevalence for each epidemiological year was estimated, starting in 2008–09.

## Results

**Fig 2:**
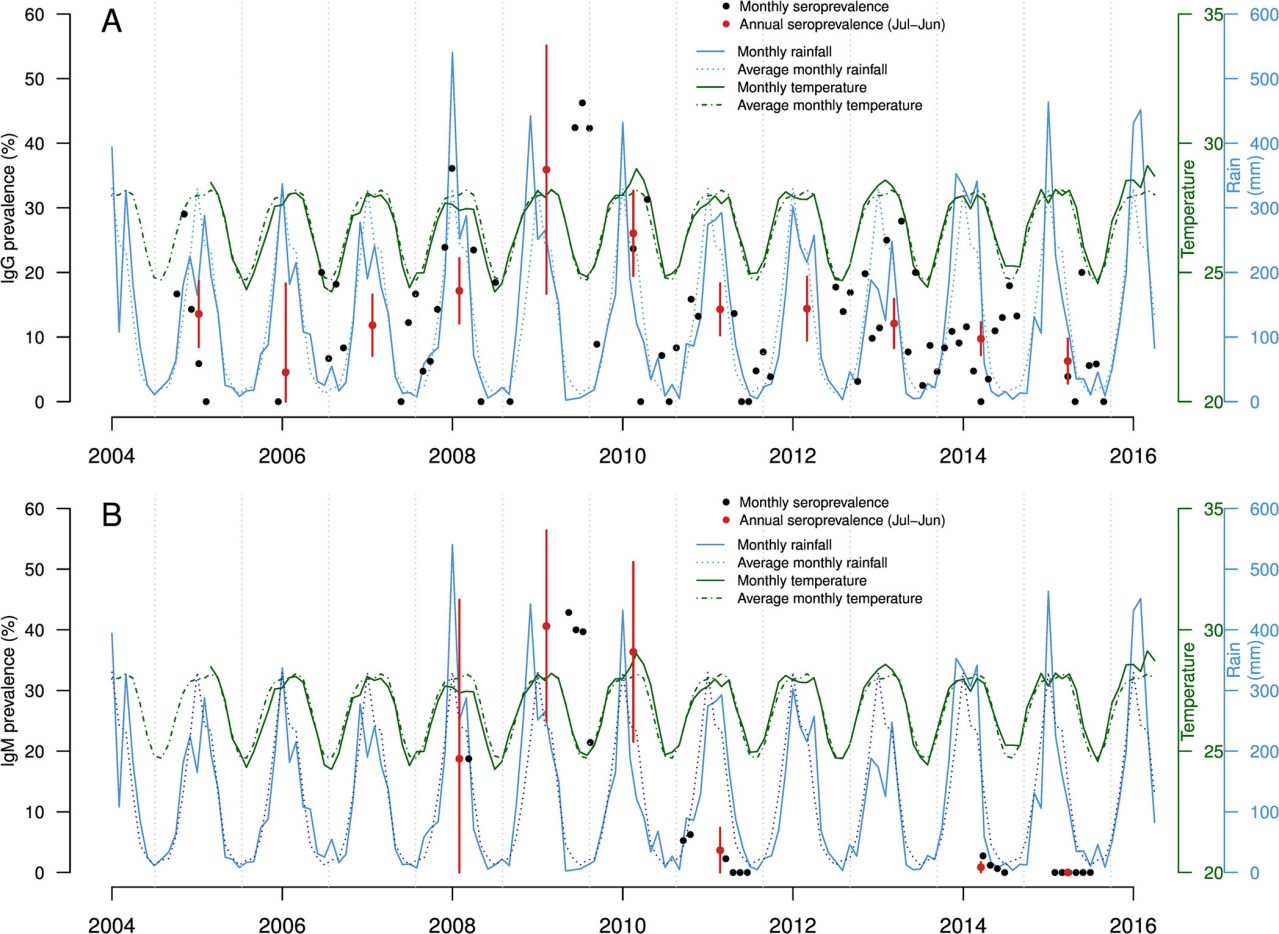
**(A) Monthly (black dots) and annual (red dots) RVF IgG prevalence and (B) IgM prevalence for the period 2004–15.** For both (A) and (B) the vertical red lines represent the 95% confidence intervals of the annual prevalences. The blue solid line represents the monthly rainfall, and the blue dashed line is the monthly rainfall values averaged over the study period (2004–15). The green solid line is the monthly mean temperature, and the green dashed line the monthly temperature values averaged over 2005–15.

**Fig 3:**
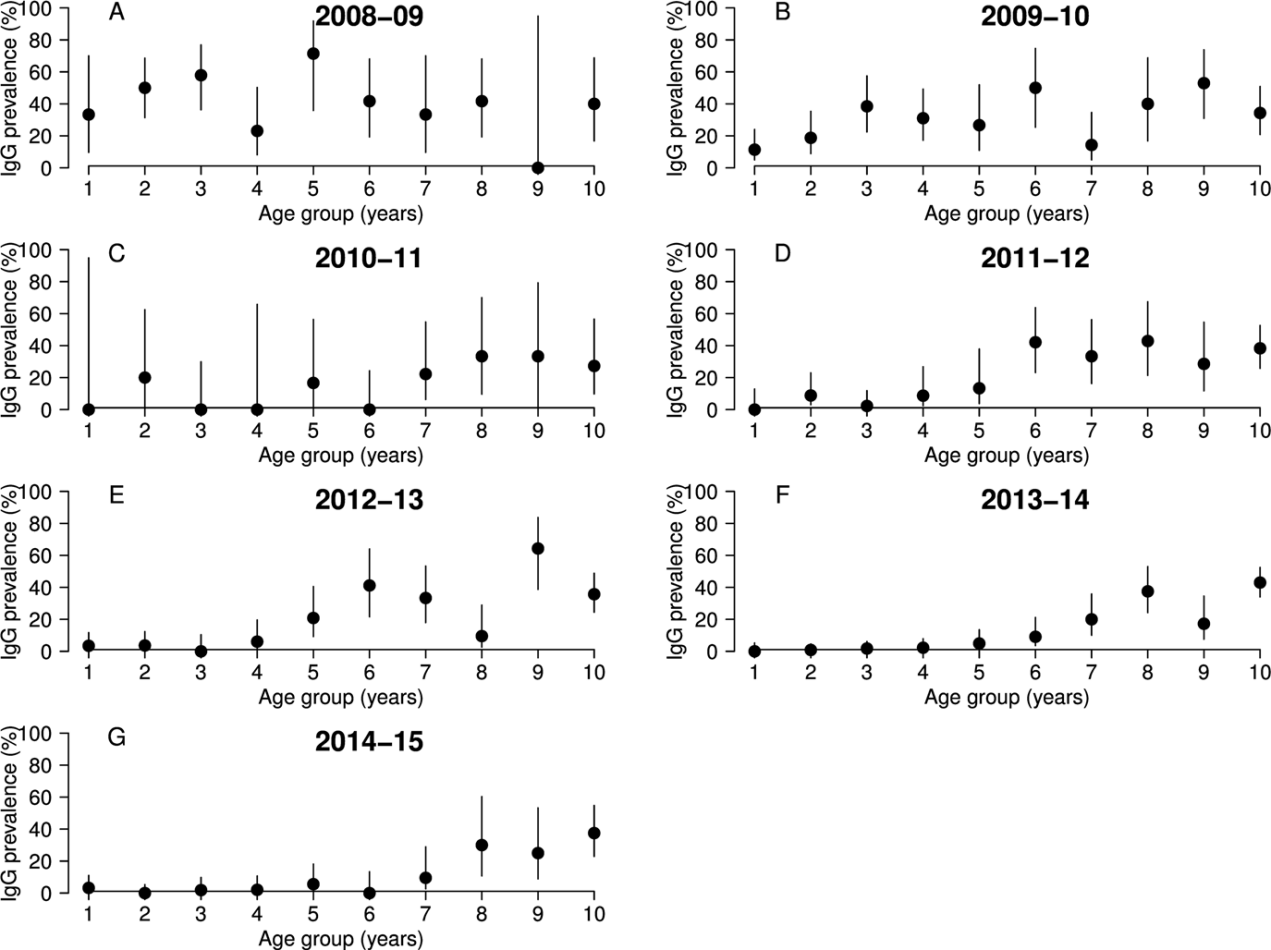
**Rift Valley fever IgG prevalence (black dots) with their 95% confidence interval (vertical black lines) per age group, for seven epidemiological years, (A) 2008–09, (B) 2009–10, (C) 2010–11, (D) 2011–12, (E) 2012–13, (F) 2013–14 and (G) 2014–15**.

After examining the annual IgG and IgM prevalences (Fig 2A and 2B, S1 Table), and IgG prevalence by age group (Fig 3A–3G, S2 Table) we divided the study period into three phases.

Phase 1 is the period with less information available. It includes the first four epidemiological years 2004–05, 2005–06, 2006–07 and 2007–08, during which IgG annual observed prevalence remained at 15% or less (Fig 2A). Newly infected animals were reported in 2007–08 (Fig 2B, IgM positive animals), but the information was scarce, and no information on the age of animals was available.

Phase 2 (2008–09 and 2009–10) suggests recent transmission of the virus. Indeed, IgG annual prevalences were significantly higher compared to the rest of the study period, reaching their maximum average value of 36% in 2008–09 (N = 142, n = 51, 95% CI [17–55]). In addition, Fig 2B shows a very high proportion of recently infected animals during that time, with IgM prevalence of 41% (N = 96, n = 39, 95% CI [25–56]) in 2008–09 and 36% (N = 77, n = 28, 95% CI [22–51]) in 2009–10. Finally, IgG prevalence was similar across all age groups in 2008–09 and 2009–10 (Fig 3A and 3B).

Phase 3 (2010–11 to 2014–15) suggests a decrease in RVF virus transmission. IgG annual prevalence was significantly lower than during phase 2, at approximately 10–15% (Fig 2A), and a steep drop in the number of new infections was observed, with IgM prevalence in 2010–11 being only 4% (N = 109, n = 4, 95% CI [0–7]) (Fig 2B). In addition, young animals (1 to 4 years old) were less affected over time, and the IgG seropositive animals were older than 5 years (Fig 3C–3G), presumably those infected in 2007–10. The 2013–14 and 2014–15 seasons suggest a very low intensity of virus transmission. IgG annual prevalence reached its lowest value in 2014–15, 6% (N = 462, n = 29, 95% CI [3–10]) (Fig 2A); and very few young animals were positive between 2012–13 and 2014–15 (Fig 3E–3G). Although no animals were IgM positive in 2014–15 (Fig 2B), two animals in the one-year old group were found IgG positive, indicating that the virus may have been still circulating (Fig 3G).

Finally, monthly rainfall pattern, and average monthly rainfall are shown in Fig 2A and 2B. Monthly rainfall varied over the study period (ranging from 2.40 to 540 mm, S3 Table). During phase 2 and in 2014–15, peaks of above-average rainfall were observed; whilst high IgM prevalence was reported in May-July 2009 during the dry season (Fig 2B). Temperature values were available for the period 2005 to 2015 (Fig 2A and 2B). Their range was narrow, with average monthly values varying between 24.7 and 28.1°C over the entire study period, and extreme monthly average minimum and maximum temperature values between 19.8°C and 32.8°C (S3 Table).

## Discussion

The analyses of serological data showed that Mayotte probably experienced an RVF epidemic in livestock around 2008–10. Peaks of above-average rainfall were observed during the epidemic phase, while variation of temperature was limited. The RVF virus seems to have remained endemic at a low level since 2011, causing few new infections. In 2015, about 95% of the livestock population was susceptible.

Serological data were collected throughout the period 2004–15, although not in a standardised manner. Retrospective data were obtained from sera stored at the Veterinary Services office. These sera were collected prior to RVF detection on the island, and it was not possible to determine exactly how this sampling was conducted in livestock. Although these results are not of a comparable value to the SESAM dataset, it gives precious information on past RVF infections, and evidence of on-going RVF virus transmission in 2008 in animals (IgM positive), shortly after the newly imported RVF strain was sequenced from humans. From 2009 onwards, farms were sampled from the CAPAM official registry [14]. Since Mayotte became a French department in 2011 and part of the EU in 2014, official registration has become compulsory, expanding the official list. The first farmers to register were possibly more affluent (with larger herds), and with an increased awareness of livestock health than those farmers who registered later. The average herd size during the study period was 7.4, and was equal to 9 in the SESAM study only. The official census from 2010 reports an average herd size of 5 [13], confirming that our sample tended to capture larger than average herds. This could have slightly biased our estimates, since animals in Mayotte are raised outdoors and therefore may share a similar exposure to mosquito bites. In addition, analyses were conducted at the scale of the island. Accounting for a smaller spatial resolution (e.g. administrative communes) would be of limited benefit for an island that is relatively small and that has a similar ecosystem throughout. Finally, in the SESAM dataset, the RVF status of an animal did not influence whether this animal would be resampled in the future. Therefore, the dataset presented is valuable to estimate RVF prevalence in Mayotte through time, especially after 2008.

Few animals were sampled in surveys 1–4 (Table 1), which resulted in large confidence intervals, giving limited knowledge for the period 2004–08 (Phase 1). The absence of age-stratified prevalence also precluded drawing any hypothesis on RVF virus transmission for that period. However, although illegal import of animals was quite common at that time, it is unlikely that all animals found positive were imported; and this supports the hypothesis that the RVF virus had been circulating on the island at least since 2004 (Phase 1), four years before the sequencing of the new virus lineage in 2008. The data in Phase 2 suggest that Mayotte experienced a large RVF epidemic in livestock; but no clinical signs as usually described in animals (abortions, high mortality in young animals) were detected, probably because no formal surveillance system in animals was in place at that time. Indeed, in humans attending the hospital for dengue-like illnesses, RVF virus was detected by RT-PCR in 8 patients between September 2007 and May 2008, confirming the presence of the virus on the island [7]. Since the implementation of the SESAM surveillance system in livestock in 2009, RVF has been monitored in livestock. There is evidence of new infections (IgM positive or one-year-old animals IgG positive); but the virus has not been detected nor isolated during Phase 3.

There is seasonality in rainfall, with very dry months in June-September, and extremely wet months, especially from December to March (average monthly rainfall from 223 to 321mm). In our dataset, however, high IgM prevalences in 2009 with IgM positive animals across the whole island were detected during the dry season. This suggested a large epidemic but does not support a direct correlation between rainfall and new cases. In other ecosystems, such as in the Horn of Africa or Southern Africa, unusually heavy rainfall or an increase in vegetation density were observed from one to six months before the emergence of new RVF cases [18–21]. Therefore, it may well be that the heavy rain observed in January-March 2009 prepared suitable conditions for mosquito breeding during the dry season, explaining the high rates of new infections in May-July 2009. In addition, field studies conducted in Mayotte in 2007 showed that natural larvae habitats specifically in rural areas allowed *Ae*. *aegypti* to survive the dry season [22]. Finally, it is also possible that a high number of new infections also occurred during the dry season of 2008 following heavy rains, but unfortunately no IgM testing was done at that time.

Very little variation was observed in temperature over the period 2005–15. During the 2010 epidemic in South Africa, temperature above 25°C was the most important risk factor [23], and experimental studies in *Culex pipiens* and *Aedes taeniorhynchus*, two RVF vector species, showed that temperature above 26°C favoured RVF virus amplification and transmission [24,25]. The high average temperatures observed in Mayotte year round may therefore provide almost constantly suitable conditions for RVF virus transmission; and RVF dynamics observed on the island maybe driven mainly by rainfall patterns.

There is no information on the virus lineage that circulated in 2004–07. The sequencing of the Mayotte 2008 lineage placed the virus into the East African clade that includes the Kenyan 2006–2007 and Madagascar 2008 lineages [9,26]. This suggests that the Mayotte 2008–10 epidemic might have followed, not only heavy rainfall, but also the import of infectious animals with an RVF virus lineage new to the Mayotte ecosystem. Trade of livestock exists from the African mainland and Madagascar, into the Comoros islands and then Mayotte (Fig 1), although the latter is illegal [27]. This import scenario was also supported by the detection of IgM positive goats illegally imported [10] from Anjouan (Fig 1), between November 2007 and March 2008. Since 2008, no virus has been isolated nor sequenced in animals. The Mayotte 2008 lineage could persist at a low level in livestock or also potentially in wildlife [1,6], causing the latest sporadic new infections in 2014 and 2015. Alternatively, as Mayotte still experiences regular animal illegal imports, introductions of other RVF virus lineages cannot be excluded, such as the Anjouan 2011 lineage detected in a zebu [28].

The working hypotheses underlying RVF virus re-emergence presented in the introduction are the concomitance of *(i)* the presence of susceptible livestock, *(ii)* an increase in vector abundance (e.g. due to heavy rainfall), and *(iii)* the presence of the virus emerging from local reservoirs or newly introduced. The data presented here suggest that Mayotte currently meets two of the conditions for re-emergence, that are: *(i)* a high proportion of susceptible livestock that reached about 95% in 2015, and (*iii*) the presence of the virus, evidenced by the new infections observed in phase 3. Therefore, we hypothesize that with heavy rainfall, such as it was observed in 2008–10, RVF virus could re-emerge. Modelling work was done to assess whether climate pattern could favor RVF virus persistence in Mayotte, which appeared to be true even under very low transmission assumption [11]. Further modelling work on RVF virus emergence can be implemented accounting for animal imports, wildlife and climate data. Different scenarios of animal imports and rainfall patterns could be tested to explain the observed epidemic dynamics and estimate the likelihood of a future epidemic. Further data collection would therefore be necessary, including ongoing climate data, surveillance in livestock, RVF prevalence in wildlife, RVF data on illegally imported animals, and virus detection, isolation, and sequencing when applicable (S1 Text).

In conclusion, this study has shown the value of repeated serological testing to explain RVF population dynamics in this island population despite limited resources. Linking these ongoing studies with additional data and modelling could also shed further light on the origin and re-emergence mechanisms of this virus.

## Supporting Information

S1 Table Annual IgG and IgM prevalences. (PDF)

S2 Table Age-stratified IgG prevalence. (PDF)

S3 Table Monthly average, minimum and maximum rainfall and temperatures over the period 2004–15 [15]. (PDF)

S1 Text Rift Valley fever surveillance in Mayotte. (PDF)

## References

[1] Bird BH, Ksiazek TG, Nichol ST, Maclachlan NJ. Rift Valley fever virus. J Am Vet Med Assoc. 2009;234:883–93. 10.2460/javma.234.7.883 19335238

[2] Daubney R, Hudson JR, Garnham PC. Enzootic hepatitis or Rift Valley fever. An undescribed virus disease of sheep, cattle and man from East Africa. J Path Bact. 1931;34:545–79.

[3] Nanyingi MO, Munyua P, Kiama SG, Muchemi GM, Thumbi S M, et al A systematic review of Rift Valley Fever epidemiology 1931–2014. Infect Ecol Epidemiol. 2015; 5:10.3402. 10.3402/iee.v5.28024 PMC4522434 26234531

[4] Linthicum KJ, Davies FG, Kairo A and Bailey CL. Rift Valley fever virus (family *Bunyaviridae*, genus *Phlebovirus*). Isolations from *Diptera* collected during an inter- epizootic period in Kenya. J Hyg Lond. 1985;95:197–209. 2862206 10.1017/s0022172400062434 PMC2129511

[5] Romoser WS, Oviedo MN, Lerdthusnee K, Patrican LA, Turell MJ, et al Rift Valley fever virus-infected mosquito ova and associated pathology: possible implications for endemic maintenance. Res Rep Trop Med. 2011;2:121–7. 10.2147/RRTM.S13947 PMC6415639 30881185

[6] Olive MM, Goodman SM and Reynes JM. The role of wild mammals in the maintenance of Rift Valley fever virus. J Wildl Dis. 2012;48:241–66. 22493102 10.7589/0090-3558-48.2.241

[7] Sissoko D, Giry C, Gabrie P, Tarantola A, Pettinelli F, Collet L, et al Rift Valley fever, Mayotte, 2007–2008. Emerg Infect Dis. 2009; 15:568–70. 10.3201/eid1504.081045 19331733 PMC2671425

[8] Lernout T, Cardinale E, Jego M, Desprès P, Collet L, Zumbo B, et al Rift valley fever in humans and animals in Mayotte, an endemic situation? PLoS One. 2013;8(9):e74192 10.1371/journal.pone.0074192 24098637 PMC3787064

[9] Cetre-Sossah C, Zeller H, Grandadam M, Caro V, Pettinelli F, Bouloy M, et al Genome analysis of Rift Valley fever virus, Mayotte. Emerg Infect Dis. 2012;18:969–71. 10.3201/eid1806.110994 22608405 PMC3358145

[10] Cetre-Sossah C, Pedarrieu A, Guis H, Defernez C, Bouloy M, Favre J, et al Prevalence of Rift Valley Fever among ruminants, Mayotte. Emerg Infect Dis. 2012;18:972–5. 10.3201/eid1806.111165 22607651 PMC3358149

[11] Cavalerie L, Charron MVP, Ezanno P, Dommergues L, Zumbo B, Cardinale E. A Stochastic Model to Study Rift Valley Fever Persistence with Different Seasonal Patterns of Vector Abundance: New Insights on the Endemicity in the Tropical Island of Mayotte. PLoS One. 2015, 10(7): e0130838 10.1371/journal.pone.0130838 26147799 PMC4493030

[12] Institut National de la Statistique et des Etudes Economiques. Résultats statistiques de Mayotte 2012 [cited 15 January 2016]. Available: http://www.insee.fr/fr/bases-de-donnees/default.asp?page=recensement/rp-mayotte/rp-mayotte.htm

[13] Ministère de l'agriculture, de l'agroalimentaire et de la forêt. 2010. Mayotte, recensement agricole. [cited 07 January 2016]. Available: http://daf.mayotte.agriculture.gouv.fr/Recensement-Agricole-2010.

[14] Chambre de l’Agriculture, de la Pêche et de l’Aquaculture de Mayotte, Service Animal. 2015. Base de données nationales d’identification.

[15] Meteo France. Le climat de Mayotte. 2012 [cited 07 Januray 2016]. Available http://www.meteo.fr/meteonet/temps/monde/prev/outremer/mayottecli.htm

[16] Meteo France. Données publiques. 2015 [cited 13 October 2015]. Available: https://donneespubliques.meteofrance.fr/.

[17] Lesnoff, M., Lancelot, R. (2012). aod: Analysis of Overdispersed Data. R package version 1.3, URL http://cran.r-project.org/package=aod

[18] Anyamba A, Chretien JP, Small J, Tucker CJ, Formenty PB, Richardson JH, et al Prediction of a Rift Valley fever outbreak. Proc Natl Acad Sci USA. 2009;106:955–9. 10.1073/pnas.0806490106 19144928 PMC2626607

[19] Anyamba A, Linthicum KJ, Small J, Britch SC, Pak E, de La Rocque S, et al Prediction, assessment of the Rift Valley fever activity in East and Southern Africa 2006–2008 and possible vector control strategies. Am J Trop Med Hyg. 2010;83(2 Suppl):43–51. 10.4269/ajtmh.2010.09-0289 20682905 PMC2913499

[20] Anyamba A, Small JL, Britch SC, Tucker CJ, Pak EW, Reynolds CA, et al Recent weather extremes and impacts on agricultural production and vector-borne disease outbreak patterns. PLoS One. 2014; 9(3):e92538 10.1371/journal.pone.0092538 24658301 PMC3962414

[21] Glancey MM, Anyamba A, Linthicum KJ. Epidemiologic and Environmental Risk Factors of Rift Valley Fever in Southern Africa from 2008 to 2011. Vector Borne Zoonotic Dis. 2015;15:502–11. 10.1089/vbz.2015.1774 26273812 PMC4545538

[22] Bagny L, Delatte H, Elissa N, Quilici S, Fontenille D. *Aedes* (Diptera: *Culicidae*) vectors of arboviruses in Mayotte (Indian Ocean): distribution area and larval habitats. J Med Entomol. 2009;46:198–207. 19351070 10.1603/033.046.0204

[23] Métras R, Jewell C, Porphyre T, Thompson PN, Pfeiffer DU, Collins LM, et al Risk factors associated with Rift Valley fever epidemics in South Africa in 2008–11. Sci Rep. 2015;5:9492 10.1038/srep09492 25804974 PMC4372659

[24] Turell MJ, Rossi CA, Bailey CL. Effect of extrinsic incubation temperature on the ability of *Aedes taeniorhynchus* and *Culex pipiens* to transmit Rift Valley fever virus. Am J Trop Med Hyg. 1985;34:1211–8. 3834803 10.4269/ajtmh.1985.34.1211

[25] Brubaker JF and Turell MJ. Effect of environmental temperature on the susceptibility of *Culex pipiens* (Diptera: *Culicidae*) to Rift Valley fever virus. J Med Entomol. 1998;35:918–21. 9835680 10.1093/jmedent/35.6.918

[26] Andriamandimby SF, Randrianarivo-Solofoniaina AE, Jeanmaire EM, Ravololomanana L, Razafimanantsoa LT, Rakotojoelinandrasana T, et al Rift Valley fever during rainy seasons, Madagascar, 2008 and 2009. Emerg. Infect. Dis. 2010;16: 963–70. 10.3201/eid1606.091266 20507747 PMC3086256

[27] Roger M, Beral M, Licciardi S, Soulé M, Faharoudine A, Foray C, et al Evidence for Circulation of the Rift Valley Fever Virus among Livestock in the Union of Comoros. PLoS Negl Trop Dis. 2014;8: e3045 10.1371/journal.pntd.0003045 25078616 PMC4117442

[28] Maquart M, Pascalis H, Abdouroihamane S, Roger M, Abdourahime F, Cardinale E, et al Phylogeographic Reconstructions of a Rift Valley Fever Virus Strain Reveals Transboundary Animal Movements from Eastern Continental Africa to the Union of the Comoros. Transbound Emerg Dis. 2014 9 12 10.1111/tbed.12267 [Epub ahead of print] 25213037

