# Supplementary material for: The Epidemiology of Rift Valley Fever in Mayotte: Insights and Perspectives from 11 Years of Data
Source: PLoS Negl Trop Dis. 2016 Jun 22;10(6):e0004783. doi: 10.1371/journal.pntd.0004783 (PMC4917248; doi:10.1371/journal.pntd.0004783)
Supplement: S1 Table — (PDF) [file pntd.0004783.s001.pdf]

S1 Table. Annual IgG and IgM prevalences (for each epidemiological year)

Epidemiological year	No. animals IgG positive	No. animals IgG tested	No. herds (clusters)	IgG prevalence	Lower CI (IgG prev)	Upper CI (IgG prev)	No. animals IgM positive	No. animals IgM tested	No. herds (clusters)	IgM prevalence	Lower CI (IgM prev)	Upper CI (IgM prev)
2004-05	33	243	67	13.58	8.46	18.70	0	0	NA	NA	NA	NA
2005-06	1	22	2	4.55	0	18.31	0	0	NA	NA	NA	NA
2006-07	20	169	46	11.83	7.08	16.59	0	0	NA	NA	NA	NA
2007-08	111	647	143	17.16	12.09	22.22	3	16	7	18.75	0	44.95
2008-09	51	142	11	35.92	16.69	55.14	39	96	9	40.62	24.86	56.39
2009-10	154	591	52	26.06	19.44	32.67	28	77	9	36.36	21.55	51.18
2010-11	26	182	20	14.29	10.26	18.32	4	109	15	3.67	0	7.37
2011-12	72	500	44	14.40	9.45	19.35	0	0	NA	NA	NA	NA
2012-13	78	645	58	12.09	8.28	15.91	0	0	NA	NA	NA	NA
2013-14	122	1253	127	9.74	7.18	12.29	5	576	70	0.87	0.01	1.73
2014-15	29	462	39	6.28	2.78	9.78	0	462	39	0	0	0

CI: Confidence Interval
