# Supplementary material for: The Epidemiology of Rift Valley Fever in Mayotte: Insights and Perspectives from 11 Years of Data
Source: PLoS Negl Trop Dis. 2016 Jun 22;10(6):e0004783. doi: 10.1371/journal.pntd.0004783 (PMC4917248; doi:10.1371/journal.pntd.0004783)
Supplement: S2 Table — (PDF) [file pntd.0004783.s002.pdf]

S2 Table. Age-stratified IgG prevalence

Age group	Epidemiological year	IgG positive	IgG tested	IgG prevalence	Lower CI	Upper CI
0-1y	2008-09	2	6	33.33	9.68	70
0-1y	2009-10	5	44	11.36	4.95	23.98
0-1y	2010-11	0	1	0	0	94.87
0-1y	2011-12	0	26	0	0	12.87
0-1y	2012-13	2	58	3.45	0.95	11.73
0-1y	2013-14	0	70	0	0	5.2
0-1y	2014-15	2	62	3.23	0.89	11.02
1-2y	2008-09	12	24	50	31.43	68.57
1-2y	2009-10	6	32	18.75	8.89	35.31
1-2y	2010-11	1	5	20	1.03	62.45
1-2y	2011-12	3	34	8.82	3.05	22.96
1-2y	2012-13	2	55	3.64	1	12.32
1-2y	2013-14	1	118	0.85	0.04	4.64
1-2y	2014-15	0	69	0	0	5.27
2-3y	2008-09	11	19	57.89	36.28	76.86
2-3y	2009-10	10	26	38.46	22.43	57.47
2-3y	2010-11	0	9	0	0	29.91
2-3y	2011-12	1	44	2.27	0.12	11.81
2-3y	2012-13	0	33	0	0	10.43
2-3y	2013-14	2	115	1.74	0.48	6.12
2-3y	2014-15	1	54	1.85	0.09	9.77
3-4y	2008-09	3	13	23.08	8.18	50.26
3-4y	2009-10	9	29	31.03	17.28	49.23
3-4y	2010-11	0	2	0	0	65.76
3-4y	2011-12	2	23	8.7	2.42	26.8
3-4y	2012-13	2	33	6.06	1.68	19.61
3-4y	2013-14	2	89	2.25	0.62	7.83
3-4y	2014-15	1	49	2.04	0.1	10.69
4-5y	2008-09	5	7	71.43	35.89	91.78
4-5y	2009-10	4	15	26.67	10.9	51.95
4-5y	2010-11	1	6	16.67	0.85	56.35
4-5y	2011-12	2	15	13.33	3.74	37.88
4-5y	2012-13	5	24	20.83	9.24	40.47
4-5y	2013-14	3	61	4.92	1.69	13.49
4-5y	2014-15	2	36	5.56	1.54	18.14
5-6y	2008-09	5	12	41.67	19.33	68.05
5-6y	2009-10	6	12	50	25.38	74.62
5-6y	2010-11	0	12	0	0	24.25
5-6y	2011-12	8	19	42.11	23.14	63.72
5-6y	2012-13	7	17	41.18	21.61	63.99
5-6y	2013-14	4	44	9.09	3.59	21.16
5-6y	2014-15	0	25	0	0	13.32
6-7y	2008-09	2	6	33.33	9.68	70
6-7y	2009-10	3	21	14.29	4.98	34.64

6-7y	2010-11	2	9	22.22	6.32	54.74
6-7y	2011-12	6	18	33.33	16.28	56.25
6-7y	2012-13	8	24	33.33	17.97	53.29
6-7y	2013-14	7	35	20	10.04	35.89
6-7y	2014-15	2	21	9.52	2.65	28.91
7-8y	2008-09	5	12	41.67	19.33	68.05
7-8y	2009-10	4	10	40	16.82	68.73
7-8y	2010-11	2	6	33.33	9.68	70
7-8y	2011-12	6	14	42.86	21.38	67.41
7-8y	2012-13	2	21	9.52	2.65	28.91
7-8y	2013-14	15	40	37.5	24.22	52.97
7-8y	2014-15	3	10	30	10.78	60.32
8-9y	2008-09	0	1	0	0	94.87
8-9y	2009-10	9	17	52.94	30.96	73.83
8-9y	2010-11	1	3	33.33	1.71	79.23
8-9y	2011-12	4	14	28.57	11.72	54.65
8-9y	2012-13	9	14	64.29	38.76	83.66
8-9y	2013-14	5	29	17.24	7.6	34.55
8-9y	2014-15	3	12	25	8.89	53.23
9-10 & >10y	2008-09	4	10	40	16.82	68.73
9-10 & >10y	2009-10	12	35	34.29	20.83	50.85
9-10 & >10y	2010-11	3	11	27.27	9.75	56.56
9-10 & >10y	2011-12	18	47	38.3	25.79	52.57
9-10 & >10y	2012-13	20	56	35.71	24.46	48.81
9-10 & >10y	2013-14	46	107	42.99	34.01	52.45
9-10 & >10y	2014-15	12	32	37.5	22.93	54.75

CI: Confidence Interval; y: years old
