# Supplementary material for: The Epidemiology of Rift Valley Fever in Mayotte: Insights and Perspectives from 11 Years of Data
Source: PLoS Negl Trop Dis. 2016 Jun 22;10(6):e0004783. doi: 10.1371/journal.pntd.0004783 (PMC4917248; doi:10.1371/journal.pntd.0004783)
Supplement: S3 Table — (PDF) [file pntd.0004783.s003.pdf]

S3 Table. Monthly average, minimum and maximum rainfall and temperatures over the period 2004-15 [*15*]

		Rainfall (mm)			Temperature (°C)		
**Season**	**Month**	**Average monthly rainfall**	**Minimum rainfall**	**Maximum rainfall**	**Average monthly**	**Average monthly minimum**	**Average monthly maximum**
Hot rainy	December	223	105.9	442.3	27.8	23.6	32.1
	January	321	165.0	540.4	27.9	24.0	31.9
	February	225	108.0	306.8	28.0	24.4	31.9
	March	232	121.3	341.4	28.1	23.9	32.8
Intermediate	April	131	58.5	257.6	27.9	23.8	32.6
	May	45	2.4	104.5	27.0	22.3	31.9
Dry cool	June	24	4.2	68.0	25.7	21.3	29.8
	July	12	5.6	25.3	24.8	20.3	29.2
	August	18	3.1	54.9	24.7	19.8	29.8
	September	24	4.9	58.6	25.3	20.6	29.9
Intermediate	October	58	12.5	110.6	26.4	21.8	31.0
	November	130	70.1	256.9	27.4	22.98	31.7
