# Supplementary material for: The Epidemiology of Rift Valley Fever in Mayotte: Insights and Perspectives from 11 Years of Data
Source: PLoS Negl Trop Dis. 2016 Jun 22;10(6):e0004783. doi: 10.1371/journal.pntd.0004783 (PMC4917248; doi:10.1371/journal.pntd.0004783)
Supplement: S1 Text — (PDF) [file pntd.0004783.s004.pdf]

S1 Text. Rift Valley fever surveillance in Mayotte

- Active surveillance: Serological surveys conducted every year in livestock (cattle, goats and sheep).

- Passive surveillance: Sudden deaths or abortions reported by farmers.
PCR analysis is conducted on every aborted female and on aborted foetus if available. If the report is done immediately and if more than one animal in the herd is affected, then serology and PCR analyses are conducted on all the animals of the herd (and abortus if applicable). If animals are PCR positive or IgM positive, then all animals are sampled a second time 2 weeks later.
- Illegal imports: IgM and IgG serology testing is done on livestock illegally imported after seizure (people are travelling with animals on small open vessels named "kwassa-kwassa") from the nearest island Anjouan (Union of the Comoros) into Mayotte (French overseas department).

Other livestock diseases are or have been monitored/tested for: Q fever, Chlamydia infection, orbiviruses infection, IBR, Neosporosis, blackleg. SESAM just recently started to cover the poultry sector in Mayotte.

The SESAM belongs more widely to the AnimalRisk regional surveillance system (http://www.animalrisk-oi.org/) that monitors emerging animal and zoonotic diseases across Indian Ocean islands (La Reunion, Seychelles, Mauritius, Comoros, Madagascar). The network recently expanded, including Mozambique and Tanzania. Animalrisk is funded by European Union, “Region Reunion” and the French government. AnimalRisk covers other livestock diseases, and diseases in other animals (poultry and pigs mainly). Depending on the area, diseases such as tickborne diseases, *Salmonella*, West Nile fever, Blue tongue and leptospirosis are being studied. In addition, this animal disease surveillance network works since 2013 in coordination with the human disease surveillance system (SEGA) with the support of the Indian Ocean Commission.
